# Assessing Social Determinants of Health Training in Graduate Medical Education: A Narrative Review Using Kirkpatrick’s Model

**DOI:** 10.7759/cureus.102981

**Published:** 2026-02-04

**Authors:** Surya L Namboodiri, Bharathi Gadad, Juan C Lopez-Alvarenga, Chelsea Chang

**Affiliations:** 1 Internal Medicine, University of Texas Rio Grande Valley School of Medicine, Edinburg, USA; 2 Psychiatry and Behavioral Sciences, University of Texas Rio Grande Valley School of Medicine, Edinburg, USA; 3 Population Health and Biostatistics, University of Texas Rio Grande Valley School of Medicine, Edinburg, USA

**Keywords:** curriculum development and evaluation, curriculum evaluation, health equity and disparities, kirkpatrick model, medical education curriculum, post graduate medical education and training, primary care education, social determinants of health (sdoh)

## Abstract

Social determinants of health (SDOH) training is a critical component of medical education, equipping physicians to address factors such as economic stability, education, and health care access. Despite its importance, the evaluation of SDOH training often focuses on lower-level outcomes, such as learner satisfaction and knowledge assessment, with limited emphasis on behavioral change and patient outcomes. Kirkpatrick’s model offers a validated framework for addressing this gap by categorizing training outcomes into four levels: Reaction, Learning, Behavior, and Results.

By applying Kirkpatrick’s model in combination with Grading of Recommendations Assessment, Development and Evaluation (GRADE) quality assessment and bibliometric network analysis, this review seeks to assess SDOH training evaluation and provide insights into designing curricula that produce measurable improvements in physician behavior and patient care outcomes.

This narrative review analyzes the efficacy of SDOH training programs in graduate medical education using studies identified in two recent scoping reviews (2019, 2024). Studies were categorized by their highest reported Kirkpatrick level: Levels 1/2 (Reaction and Learning) or Levels 3/4 (Behavior and Results). If a study spanned multiple levels, it was categorized at the highest level. This review further uniquely integrates GRADE and bibliometric co-authorship network analysis to explore evidence quality and author collaborations.

A total of 33 studies were analyzed, with 79% classified as Levels 1/2 and 21% as Levels 3/4. Most studies used Level 1 evaluation methods, primarily subjective surveys. Among Level 3/4 studies, 71% used career tracking as a proxy for behavioral change, and only three assessed patient outcomes (Level 4). Most studies also reported positive results, with two noting alternate outcomes related to program length and parental trust.

GRADE analysis rated all studies as low or very low quality, reflecting the predominance of observational or quasi-experimental designs without randomization or blinding. Only two studies incorporated control groups but still received low ratings due to high risk of bias and lack of replication. Bibliometric co-authorship network analysis identified three distinct institutional clusters of collaboration: one centered around Cincinnati Children’s Hospital (2014-2018), one around Montefiore Medical Center (2010-2012), and a dyad at Johns Hopkins Hospital (2016).

Most SDOH training evaluations focused on Kirkpatrick Levels 1/2, offering limited insight into real-world impact. While Kirkpatrick’s framework helps organize outcomes, it is limited, as interventions with multifactorial effects may not directly link to training. Consistently low GRADE ratings and limited collaboration across institutions highlight the need for stronger study designs and more coordinated research efforts.

Most SDOH training evaluations rely on subjective outcomes and are supported by low-quality evidence, limiting conclusions about impact on clinical practice. Without rigorous, higher-level evaluation, the true impact of SDOH training in graduate medical education on clinical practice and health equity remains uncertain. Advancing SDOH education will require longitudinal curricula, objective and patient-centered outcomes, and institutionally supported, multi-site study designs to generate generalizable evidence.

## Introduction and background

Social determinants of health (SDOH) can be defined as non-medical factors such as place of birth, education, built environment, and access to money and resources that play a powerful role in influencing health outcomes [1]. Specifically, SDOH can contribute to health inequity by perpetuating systematic and avoidable differences in access and availability of quality health care. Therefore, recognizing and addressing SDOH is considered a critical competency for physicians [2]. Training in SDOH has thus emerged as an essential component of medical education, aimed at equipping physicians with the skills needed to understand, address, and advocate for patients' social needs [3].

Despite widespread acknowledgment of its importance, the development, implementation, and evaluation of SDOH curricula face significant challenges. Current literature highlights a range of educational approaches [4,5]. However, the evaluation methods used to measure the efficacy of these approaches show significant heterogeneity and poor structure [6,7]. As a result, establishing the true effectiveness of individual training programs remains a major limitation in advancing SDOH education.

Current assessments of SDOH training predominantly focus on low-level outcomes such as learner satisfaction or self-reported changes in knowledge and attitudes [8]. Few studies utilize rigorous frameworks to evaluate the impact of these trainings on higher-order outcomes such as behavioral change, clinical practice, or patient outcomes. The reliance on self-reported data and short-term metrics limits the ability to draw meaningful conclusions about the long-term outcomes of these programs, highlighting a need for a more structured evaluation approach.

Kirkpatrick’s model of training evaluation provides a validated structure for addressing these evaluation gaps [9]. This model categorizes learning outcomes into four levels: Reaction (learner satisfaction), Learning (knowledge acquisition), Behavior (application of skills in practice), and Results (impact on patient outcomes and organizational goals). Applying this model to assess SDOH training offers a more comprehensive approach to understanding efficacy. By emphasizing higher-order outcomes, such as behavioral changes and patient-centered metrics, this framework can help address the limitations identified in existing reviews and advance the field toward evidence-based educational practices.

Given these challenges and opportunities, this literature review aims to analyze the efficacy of various SDOH training programs in graduate medical education by novelly applying Kirkpatrick’s model to an existing body of literature. This research additionally seeks to provide further insights into designing and implementing effective SDOH educational programs that produce measurable improvements in clinical practice through Grading of Recommendations Assessment, Development and Evaluation (GRADE) and bibliometric analyses.

## Review

Methods 

This structured narrative review explored the efficacy of SDOH training programs in graduate medical education by utilizing studies identified in two recent scoping reviews [8,10]. These scoping reviews comprehensively summarize existing literature on SDOH curricula and their evaluation methods, providing a foundational body of work for further analysis.

Study Selection Criteria  

Studies included in this analysis were drawn exclusively from North American programs to ensure relevance to the region. Inclusion criteria required that studies evaluate SDOH curricula in graduate medical education, with explicit assessment methods reported. Explicit assessment methods refer to evaluation approaches that are clearly described in the study, allowing the type, scope, and metrics of the SDOH training evaluations to be readily understood from the article. Studies were excluded if they lacked evaluative data or originated outside North America to account for regional variability in medical education structure.

Study Categorization  

To simplify the analysis and align with Kirkpatrick’s framework, studies were grouped into two categories based on their highest reported level of assessment:

Levels 1 and 2 (reaction and learning): Studies assessing subjective learner satisfaction, self-reported knowledge, or attitudes were classified as Level 1. Studies measuring objective learning and knowledge acquisition were classified as Level 2.

Levels 3 and 4 (behavior and results): Studies evaluating behavioral changes were classified as Level 3. Studies evaluating clinical practice improvements or patient outcomes were classified as Level 4.

If a study reported outcomes across multiple levels, it was categorized according to the highest level achieved. For instance, a study evaluating both learner satisfaction (Level 1) and behavioral changes (Level 3) would be assigned to the Levels 3/4 category. Additionally, any level beyond Reaction (Level 1) must display objective measurements.

Data Extraction, Analysis, and Quality Assessment

Key data points, including participants, duration, and assessment types, were extracted for each study, and studies were categorized using Kirkpatrick’s model. The quality of evidence was evaluated using the GRADE method to assess each article based on study design, risk of bias, consistency, directness, and precision of reported outcomes [11]. Bibliometric analysis was conducted using Scopus, which provided a comprehensive database of the included studies. The Scopus data were then imported into VOSviewer to visualize co-authorship networks and examine connections between studies. Additionally, qualitative thematic analysis was performed to identify recurring strengths and limitations in assessment methodologies.

Results 

A total of 33 studies were included, as shown in Table 1, which describes each article, including evaluation methods, Kirkpatrick categorization, and GRADE analysis.

**Table 1:** Summary of 33 studies evaluating SDOH training. SDOH: Social determinants of health; OB/GYN: Obstetrics and gynecology; UCSF: University of California, San Francisco.

PMID	Author, Year	Institution	Participants who completed evaluations	Trainee type	Duration of intervention	Evaluation methods	Highest Kirkpatrick level	GRADE quality of evidence
29190406	Noriea et al. (2017) [12]	University of Alabama at Birmingham	16	Internal Medicine Residents	1 year	Pre- and/or post-intervention surveys	1	Very low
28145945	Basu et al. (2017) [13]	Cambridge Health Alliance	17	Internal Medicine Residents	1 year	Pre- and/or post-intervention surveys; Knowledge testing/application	2	Very low
20795806	Klein and Vaughn (2010) [14]	Cincinnati Children’s Hospital Medical Center	33	Pediatrics Residents	1 day	Written reflections/verbal discussions	1	Very low
18162745	Gregg et al. (2008) [15]	Oregon Health & Science University	Not reported	Internal Medicine Residents	8 days	Pre- and/or post-intervention surveys; Written reflections/verbal discussions	1	Very low
30633798	Jacobs et al. (2019) [16]	Saint Louis University Family Medicine Residency	22	Family Medicine Residents	3 years	Pre- and/or post-intervention surveys; Written reflections/verbal discussions; Knowledge testing/application	2	Very low
12269530	Eddy and Labuguen (2002) [17]	Virginia Commonwealth University	13	Family Medicine Residents	1 year	Pre- and/or post-intervention surveys	1	Very low
33557815	Bradley et al. (2021) [18]	The Veterans Health Administration Hospital, Vermont	12	Internal Medicine Residents	3 days	Written reflections/verbal discussions	1	Very low
33058502	Daya et al. (2021) [19]	San Francisco Tertiary Hospital	66	Internal Medicine Clerkship – Medical Students	2 months	Written reflections/verbal discussions	1	Very low
32105356	Goroncy et al. (2020) [20]	The Christ Hospital/University of Cincinnati	34	Family Medicine Residents	3 years	Written reflections/verbal discussions	1	Very low
31948434	Gard et al. (2020) [21]	Northwestern University	129	Internal Medicine and Family Medicine Residents	2 months	Pre- and/or post-intervention surveys	1	Very low
33816790	Ramadurai et al. (2021) [22]	University of Colorado	11	Internal Medicine, Family Medicine, Emergency Medicine Residents	3 years	Pre- and/or post-intervention surveys	1	Very low
33391594	Christmas et al. (2020) [23]	Johns Hopkins Bayview Medical Center	94	Internal Medicine Residents	2–8 weeks	Pre- and/or post-intervention surveys	1	Very low
34872529	Morrison et al. (2021) [24]	Johns Hopkins All Children’s Hospital Center for Simulation	13	Pediatrics Residents	1 day (follow-up at 1 year)	Pre- and/or post-intervention surveys; Written reflections/verbal discussions	1	Very low
34172296	Mullett et al. (2022) [25]	University of Washington	109	Pediatrics Residents	1 year	Pre- and/or post-intervention surveys	1	Very low
34917754	Traba et al. (2021) [26]	Rutgers New Jersey Medical School	61	Pediatrics Residents	1 year	Pre- and/or post-intervention surveys	1	Very low
32743065	Balighian et al. (2020) [27]	Johns Hopkins University Pediatric and Medicine	29	Pediatrics Residents	4 weeks	Pre- and/or post-intervention surveys	1	Very low
24011747	Tschudy et al. (2013) [28]	Johns Hopkins Hospital	50	Pediatrics Residents	1 year	Pre- and/or post-intervention surveys	1	Very low
23997880	Sufrin et al. (2012) [29]	San Francisco County Jail	9	OB/GYN Residents	6 weeks	Pre- and/or post-intervention surveys	1	Very low
31267340	Lazow et al. (2019) [30]	Cincinnati Children’s Hospital Medical Center	19	Pediatrics Residents	1 day	Pre- and/or post-intervention surveys; Knowledge testing/application	2	Very low
29684581	Lazow et al. (2018) [31]	Cincinnati Children’s Hospital Medical Center	21	Pediatrics Residents	1 day	Pre- and/or post-intervention surveys	1	Very low
31312721	Lax et al. (2019) [32]	Children’s Hospital at Montefiore	55	Pediatrics Residents	9 months	Pre- and/or post-intervention surveys	1	Very low
30005114	Lochner et al. (2018) [33]	University of Wisconsin	120	Family Medicine Residents	3 years	Pre- and/or post-intervention surveys	1	Very low
30800848	Schmidt et al. (2017) [34]	Emory University School of Medicine	19	Internal Medicine Residents	4 weeks	Pre- and/or post-intervention surveys; Written reflections/verbal discussions	1	Very low
35353034	Connors et al. (2022) [35]	University of Alberta, Canada	35	Pediatrics Residents	4 weeks	Written reflections/verbal discussions	1	Very low
12815083	Jacobs et al. (2003) [36]	Cook County Hospital	Not reported	Internal Medicine and Pediatrics Residents	4 days	Pre- and/or post-intervention surveys	1	Very low
27480423	Real et al. (2016) [37]	Cincinnati Children’s Hospital Medical Center	37	Pediatrics Residents	3 days over 3 months	Pre- and/or post-intervention surveys; Patient outcomes	4	Very low
27988207	Real et al. (2017) [38]	Cincinnati Children’s Hospital	44	Pediatrics Residents	2 weeks	Pre- and/or post-intervention surveys; Knowledge testing/application; Behavior change; Patient outcomes	4	Very low
24602579	Klein et al. (2014) [39]	Cincinnati Children’s Hospital Medical Center	47	Pediatrics Residents	2 days	Behavior change; Patient outcomes	4	Low
20703151	Kuo et al. (2010) [40]	University of California, San Francisco	37	Pediatrics Residents	3 years	Pre- and/or post-intervention surveys; Behavior change	3	Very low
21240789; 18367900	Fornari et al. (2011) and Strelnick et al. (2008) [41,42]	Montefiore Medical Center and Albert Einstein College of Medicine	110	Internal Medicine, Family Medicine, and Pediatrics Residents	3 years	Pre- and/or post-intervention surveys; Written reflections/verbal discussions; Knowledge testing/application; Behavior change	3	Very low
16520503	Furin et al. (2006) [43]	Brigham and Women’s Hospital, Boston	6	Internal Medicine Residents	4 years	Knowledge testing/application; Behavior change	3	Very low
31413996	Knox et al., (2018) [44]	University of Wisconsin	16	Internal Medicine Residents	3 years	Pre- and/or post-intervention surveys; Knowledge testing/application; Behavior change	3	Very low
22399566	O’Toole et al. (2012) [45]	Cincinnati Children’s Hospital Medical Center	40	Internal Medicine and Pediatrics Residents	5 months	Pre- and/or post-intervention surveys; Behavior change	3	Very low

**Figure 1:**
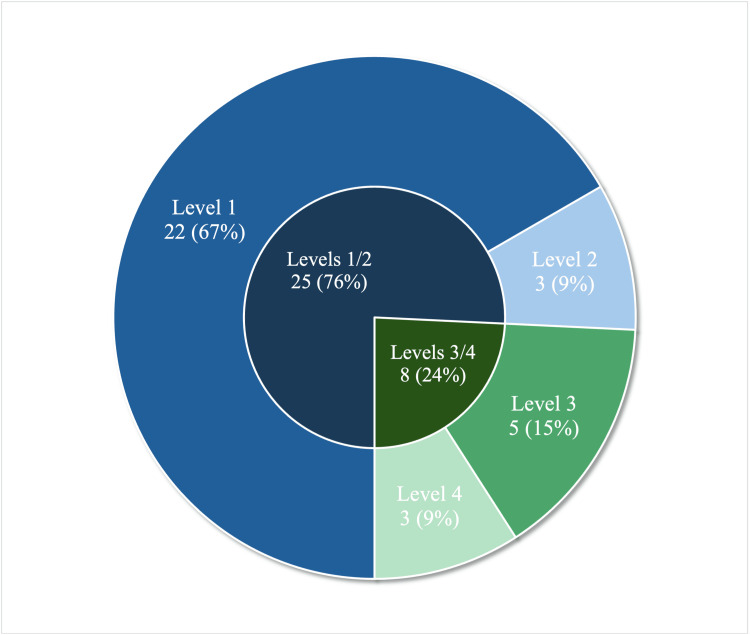
Distribution of 33 studies across Kirkpatrick levels. Visualization of the distribution of 33 studies across the four Kirkpatrick levels, with most conducted at Levels 1/2 and fewer at Levels 3/4. Results are labeled as N (%), where N indicates the number of studies whose highest categorization reached that level, and % indicates the corresponding percentage.

Twenty-five studies were categorized as Levels 1 or 2 [12-36]. Eight studies were categorized as Levels 3 or 4 [37-45]. These findings are graphically visualized in Figure 1.

**Figure 2:**
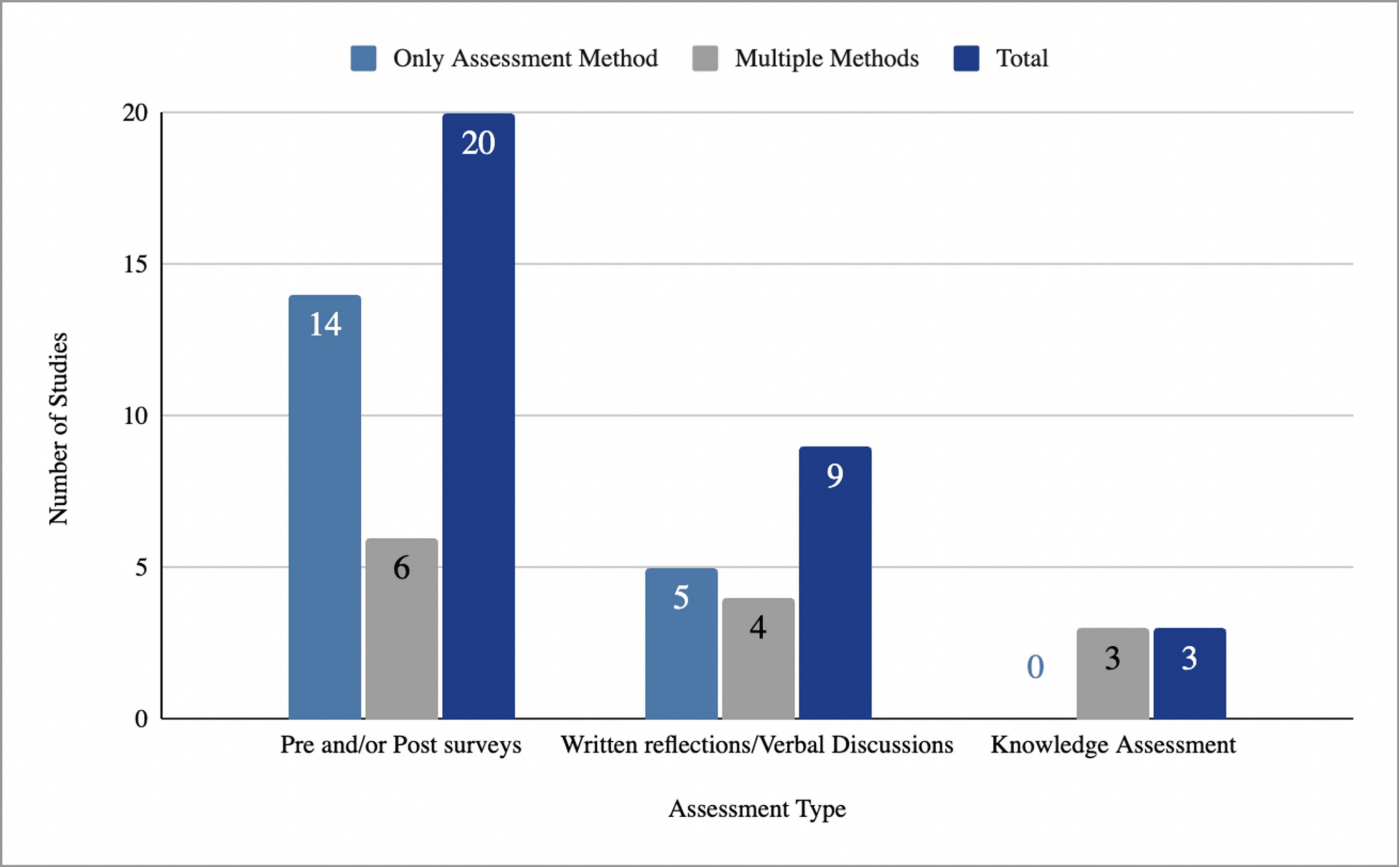
Assessment methods used in the 25 studies categorized as Levels 1/2. Number of studies using different assessment methods in Levels 1/2 studies, stratified by whether the method was used alone or in combination with others. Pre- and/or post-intervention surveys were most frequently reported.

When subdividing the Levels 1/2 studies further, we found that 22 studies utilized only Level 1 evaluation methods, such as pre- and post-intervention surveys and written reflections/open discussions [12,14,15,17-29,31-36], and three studies assessed knowledge acquisition objectively [13,16,30]. Surveys were the most commonly used evaluation tool, appearing in 20 Level 1 studies: 14 as the sole method and six in combination with other approaches [12,13,15-17,21-34,36]. Nine studies categorized as Level 1 used written reflections/open discussions to evaluate participants, with five using this method alone and four using a combination of methods [14-16,18-20,24,34,35]. Overall, evaluation strategies at this level were predominantly subjective and focused on learner reactions rather than objective measures of learning. Results are summarized in Figure 2.

**Figure 3:**
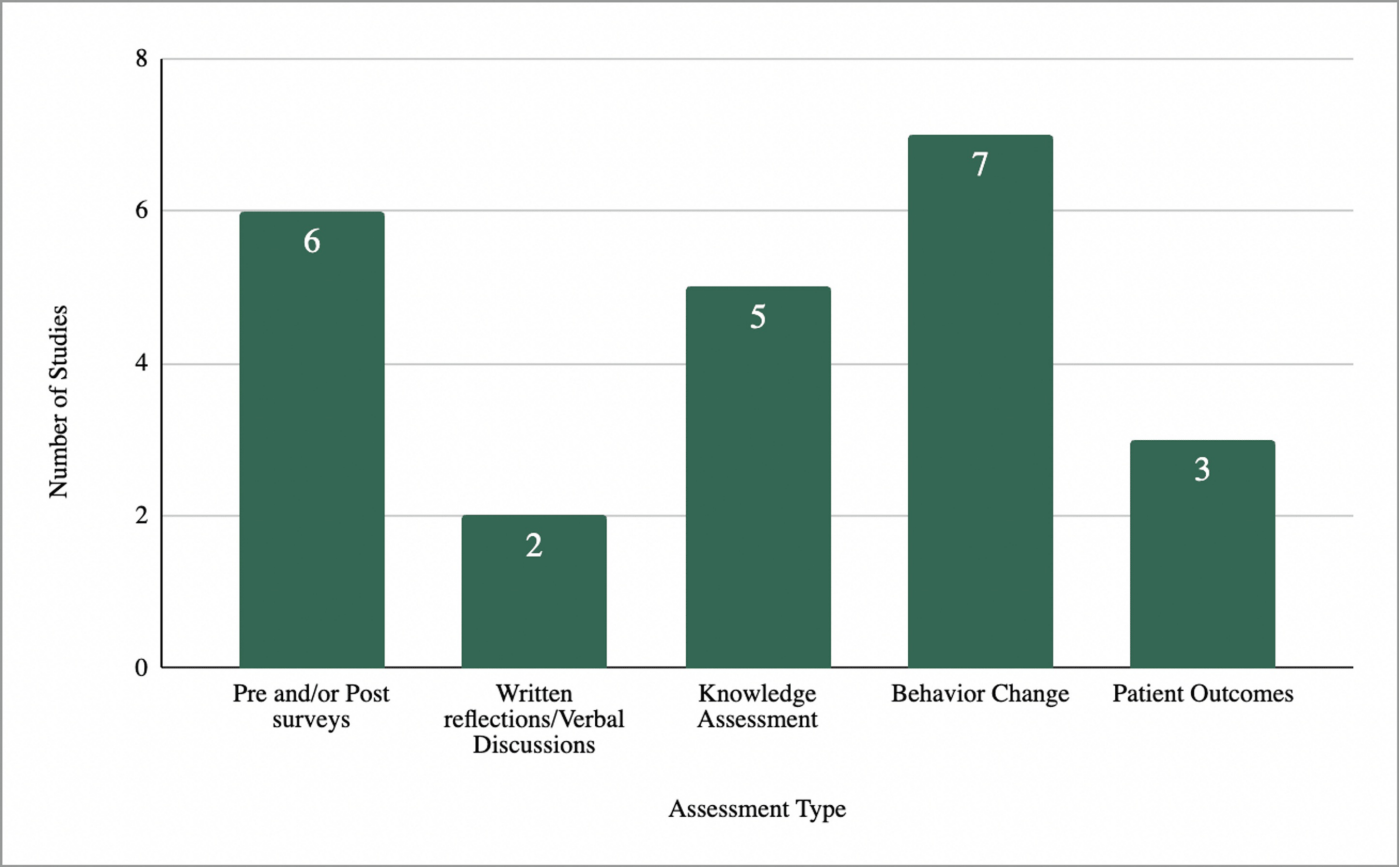
Assessment methods used in the eight studies categorized as Levels 3/4. Distribution of assessment methods used in Levels 3/4 studies. Bars indicate the number of studies using each method, with behavior change being the most frequently reported.

Of the studies categorized within Levels 3/4, seven of the eight studies measured behavioral change [38-45]. Five of these seven studies measured career tracking of postgraduate work as a proxy for behavioral change [38,40-44]. Two of these seven studies more directly measured behavioral change through resource referral in one and faculty assessment in another, both relatively uncommon measures [39,45]. Three of the seven studies measured results related to patient outcomes (Kirkpatrick Level 4), as shown in Figure 3 [37-39]. These results were ascertained based on family feedback through caregiver or parent responses to resident treatment. One study placed in the Level 1 category used this framework and claimed to have assessed behavioral change, but did not truly do so, as results were subjective to participants [24]. Higher-level evaluations remain uncommon and are largely inferred through indirect proxies rather than measured through practice-based or patient-centered outcomes.

Thirty-one of the 33 studies primarily reported positive outcomes of their programs. Of the two studies that reported alternate outcomes, one was in the Levels 1/2 category and reported short program length as a cause of lower resident satisfaction, and the other was in the Levels 3/4 category and found no difference in one of their measured outcomes (trust/respect of parents of pediatric patients) [36,39].

GRADE analysis showed that all 33 studies were considered low or very low quality. All studies were observational or quasi-experimental, placing them at baseline low quality. None of the studies met criteria for upgrading in quality, as they lacked randomization, blinding, or large, direct, and consistent effects that would reduce the risk of bias. All studies were single-center reports without validated replication. Two studies reported using control groups, but due to risk of bias and inconsistency from lack of replication, they still received low or very low quality classifications [30,38]. One study received a low classification rather than very low due to an observational study design with a control group and objective outcomes with measured statistical significance (Table 1) [38].

**Figure 4:**
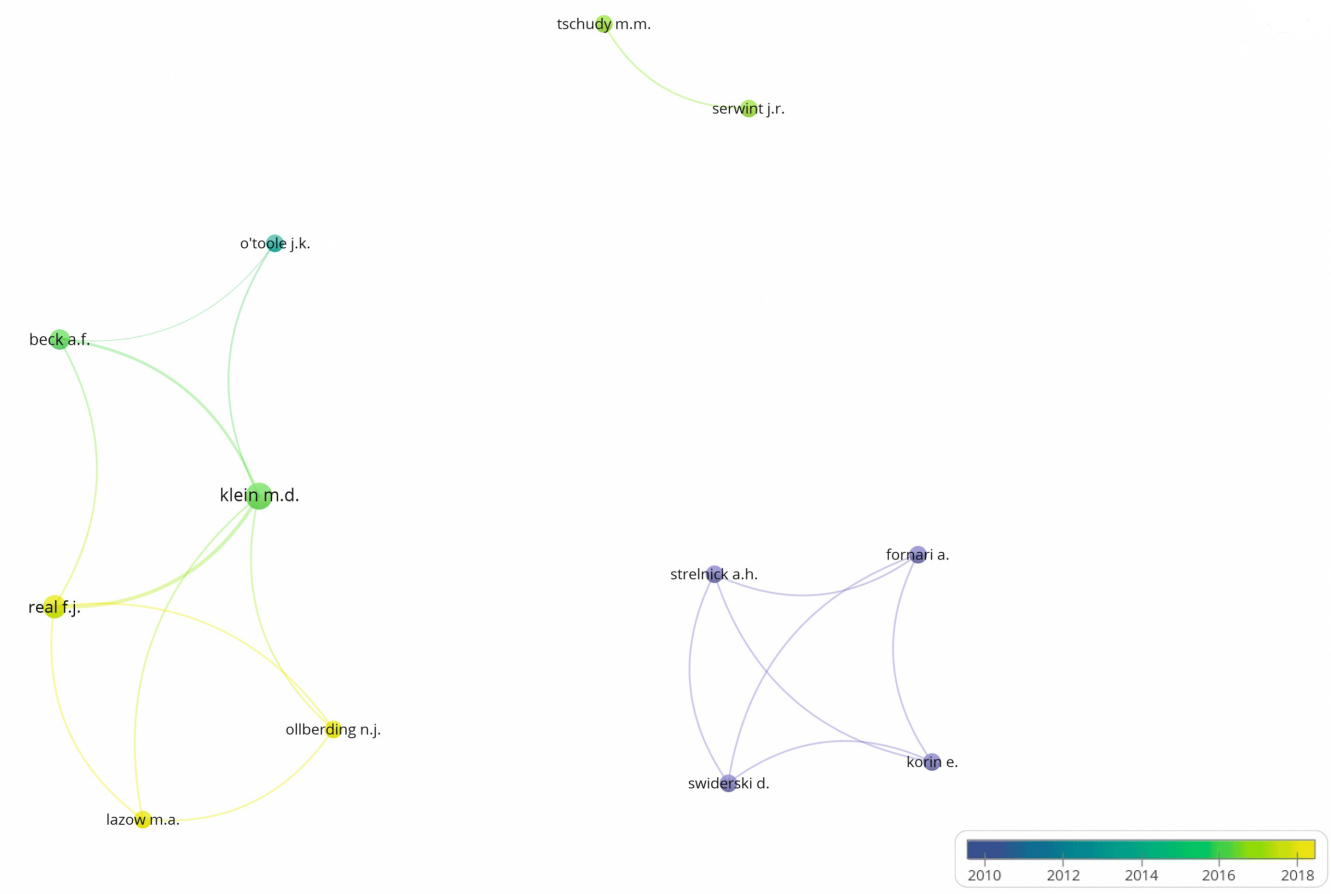
Bibliometric co-authorship network analysis. Visualization of the bibliometric co-authorship network generated using VOSviewer, illustrating clusters of researchers and their collaborative connections. Node size reflects the number of publications by each author (a larger node indicates more publications), while node color represents the average year of publication (blue = earlier, yellow = more recent). The thickness of the connecting lines indicates the strength of co-authorship links, with thicker lines indicating more frequent collaboration.

Bibliometric co-authorship network analysis identified three distinct clusters of collaboration among the authors of the included studies. The first cluster consisted of six authors, with Klein M et al. serving as the core author, demonstrating strong connections with collaborators such as Lazow MA, Ollberding NJ, Real FJ, O'Toole JK, and Beck AF. This cluster’s publications ranged from approximately 2014 to 2018 and were affiliated with Cincinnati Children’s Hospital. The second cluster comprised four authors, Swiderski D, Strelnick AH, Fornari A, and Korin E, who showed dense co-authorship connections primarily around 2010-2012. This cluster was affiliated with Montefiore Medical Center. The third cluster included two authors, Serwint JR and Tschudy MM, who collaborated with each other in 2016. This dyad was affiliated with Johns Hopkins Hospital. There were no connections between these clusters, indicating distinct and independent groups within the overall co-authorship network. The depiction of results can be seen in Figure 4 via VOSviewer.

Two studies were grouped as one entity, as they referred to the same intervention [41,42]. Three additional studies from the original scoping reviews were removed based on exclusion criteria. One study was included only once instead of twice because it overlapped between both scoping reviews [37]. Another study did not provide enough information about evaluation methods [46]. Lastly, another study was conducted outside North America and was also excluded from our analysis per the inclusion criteria [47].

Discussion 

The vast majority of studies categorized fell into Levels 1/2 of Kirkpatrick’s evaluation model. This predominance of reaction- and perception-based assessments aligns with prior literature and likely reflects structural and methodological constraints rather than a lack of curricular intent. Common barriers include short intervention duration, limited access to longitudinal learner or patient data, challenges in attributing downstream outcomes to a single educational exposure, and a lack of institutional incentives to pursue higher-level evaluation. While reaction-level assessments provide insight into learner satisfaction and perceived relevance, they offer limited evidence of educational effectiveness and are unable to demonstrate meaningful change that benefits physicians-in-training or patient outcomes, despite what results may claim.

Although prior scoping reviews reported frequent knowledge-based assessments, this review found that most such assessments relied on subjective self-report rather than objective measurement. While this finding is not unexpected, it has important implications for validity, as perceived knowledge gain may not accurately reflect acquisition, retention, or application. Reliance on subjective measures additionally limits comparability across programs and weakens conclusions regarding intervention effectiveness. Thus, there is a clear need for validated and performance-based assessments in SDOH education research.

Studies reporting behavioral change most often relied on career tracking as a proxy outcome. While career choice may reflect cumulative educational exposure, it is inherently multifactorial and distant from individual interventions, making attribution to a specific SDOH curriculum problematic. As a result, many studies classified as Level 3 may overstate evidence of behavioral change, as they lack direct measures linking curricular participation to clinical behaviors or decision-making. Although one study found an association between program participation and pursuit of primary care careers [39], causality remains limited by confounding factors and study design.

GRADE analysis showed that all studies were rated as low or very low quality, reflecting a consistent pattern of methodological limitations across the included literature and underscoring the low certainty of evidence supporting SDOH training interventions. Many studies relied heavily on subjective, self-reported outcomes without standardized or validated assessment tools, increasing the risk of response and social desirability bias and limiting comparability across programs. The predominance of observational designs, while practical in educational settings, further restricts causal inference. The absence of randomization, blinding, and multi-site replication compounded these biases and reduced the generalizability of findings. Even studies that included control groups were limited by selection bias and single-site design. The single study rated as low quality demonstrated relatively stronger internal validity using objective, statistically supported outcome measures, though it still lacked sufficient methodological rigor [39]. Overall, these findings highlight the need for more robust, standardized, and replicable study designs to strengthen the evidence base and support broader adoption of effective SDOH educational interventions.

The bibliometric analysis demonstrated that authorship networks were largely siloed within individual institutions, with limited connections between different centers. The largest and most recent cluster was from Cincinnati Children’s Hospital and could serve as a hub for broader collaboration. In contrast, the Montefiore Medical Center cluster showed earlier activity with fewer recent publications, while the Johns Hopkins Hospital dyad remained isolated from other groups. This lack of collaboration highlights a missed opportunity for replication and multi-site studies, perpetuating weaker study designs and poor quality [48,49]. These findings also suggest an opportunity to build stronger inter-institutional partnerships and expand representation beyond these few institutions.

We further identified that, among participants, the majority were from primary care disciplines. Only two studies included residents from non-primary care specialties, OB/GYN and emergency medicine [21,28]. This imbalance may reflect under-implementation or underreporting of SDOH curricula outside primary care, despite the universal relevance of social determinants across all clinical specialties. Expanding both training and research efforts across diverse disciplines is therefore essential to ensure comprehensive physician preparedness. Additionally, as educational interventions are shaped by various contextual factors such as learner needs, training stage, geographical setting, and educational environment, uniform approaches to curriculum design and evaluation are unlikely to be effective across all settings [50-52]. Thus, tailoring educational strategies to context is necessary for meaningful and sustainable outcomes.

The thematic analysis showed that most studies reported positive outcomes, but these findings should be interpreted with caution. Publication bias likely contributes to the high number of favorable results, especially when outcomes are based on self-reported measures without objective validation. The lack of neutral or negative findings may overestimate the effectiveness of SDOH curricula and limit opportunities for improvement. Addressing this bias will require clearer outcome reporting, inclusion of null results, and greater use of objective and patient-centered measures.

Some limitations of this research include the methodology and sample size, in that we included only studies within two scoping reviews, which may introduce selection bias in study inclusion and limit comprehensiveness, as newer studies (post-2024) were not included. This methodology was intentionally selected to efficiently synthesize the most up-to-date literature on SDOH training and to build upon prior work, specifically by performing analyses on an established body of research. Another limitation is that we had only one reviewer for the studies. Having more reviewers in the future could confirm classifications and strengthen results and conclusions. A further limitation is the inclusion of one study conducted with medical students rather than residents in our analyses, as medical students may have limited ability to contribute to outcome-based measures. However, we chose to keep this study in our analysis as it reflects an important segment of participants in SDOH training curricula [53]. Another limitation to the generalizability of our review is the exclusion of non-North American studies. This choice was made due to differences in medical education structure between regions; however, this limitation underscores the need for expanded, high-quality global research to examine how SDOH training is designed and evaluated across diverse health systems [54]. Only one study was excluded based on this criterion; thus, its exclusion is unlikely to meaningfully alter the overall findings in our review. Lastly, another limitation of this review is the use of Kirkpatrick’s model itself as a tool for analysis of SDOH training research. In SDOH education, Kirkpatrick’s model provides limited insight into how non-curricular factors influence higher-level outcomes.

Future SDOH educational research should move beyond subjective evaluations and focus on identifying curriculum components that enable higher-level outcomes, including addressing context as stated earlier [50-52]. Emerging evidence also suggests that programs with a longitudinal structure, clear emphasis on SDOH competencies, and dedicated faculty leadership are more likely to achieve higher Kirkpatrick levels [55]. These findings support the development of standardized, performance-based assessments that directly measure clinical behaviors, decision-making, and patient-facing outcomes. Multi-site, longitudinal study designs are particularly needed to improve generalizability and allow attribution of outcomes to specific curricular elements. An example of this approach is the Alliance for Academic Medicine-funded SDOH initiative at the UTRGV School of Medicine Student Run Clinic in Las Penitas, Texas, which directly measures patient outcomes using validated screening tools for diabetes, hypertension, and colorectal cancer. Programs of this type demonstrate the feasibility of aligning SDOH education with measurable clinical endpoints and offer a model for future curriculum development and evaluation in graduate medical education.

## Conclusions

Most published SDOH training studies rely on low-level Kirkpatrick outcomes and subjective assessments, limiting the strength and generalizability of their conclusions. Consistently low GRADE ratings reflect systemic methodological weaknesses, including single-site study designs, limited objective measurement, and insufficient linkage between curricular interventions and downstream clinical outcomes. To advance SDOH education, accrediting bodies and training institutions should incentivize longitudinal curricula, require the use of objective and patient-centered outcome measures, and support multi-institutional collaboration to enable higher-level evaluation. Embedding SDOH competencies into accreditation standards and program evaluation frameworks may further promote accountability and consistency across training programs. Strengthening methodological rigor and inter-institutional collaboration will also be essential to generating actionable evidence and ensuring that SDOH education translates into meaningful improvements in clinical practice and health equity.
